# The Effect of Syntactic Impairment on Errors in Reading Aloud: Text Reading and Comprehension of Deaf and Hard of Hearing Children

**DOI:** 10.3390/brainsci10110896

**Published:** 2020-11-23

**Authors:** Ronit Szterman, Naama Friedmann

**Affiliations:** 1 Language and Brain Lab, Sagol School of Neuroscience and School of Education, Tel Aviv University, Tel Aviv 699787, Israel; ronitszt@tauex.tau.ac.il; 2 Division of Special Education, Ministry of Education Israel, Jerusalem 9510402, Israel

**Keywords:** deaf and hard of hearing, syntax, reading aloud, reading comprehension, dyslexia

## Abstract

Deaf and Hard of Hearing (DHH) children show difficulties in reading aloud and comprehension of texts. Here, we examined the hypothesis that these reading difficulties are tightly related to the syntactic deficit displayed by DHH children. We first assessed the syntactic abilities of 32 DHH children communicating in spoken language (Hebrew) aged 9;1–12;2. We classified them into two groups of DHH children—with and without a syntactic deficit according to their performance in six syntactic tests assessing their comprehension and production of sentences with syntactic movement. We also assessed their reading at the single word level using a reading aloud test of words, nonwords, and word pairs, designed to detect the various types of dyslexia, and established, for each participant, whether they had dyslexia and of what type. Following this procedure, 14 of the children were identified with a syntactic deficit, and 15 with typical syntax (3 marginally impaired); 22 of the children had typical reading at the word level, and 4 had dyslexia (3 demonstrated sublexical reading). The main experiment examined reading aloud and comprehension of 6 texts with syntactic movement (which contained, e.g., relative clauses and topicalized sentences), in comparison to 6 parallel texts without movement. The results indicated a close connection between syntactic difficulties and errors in reading aloud and in comprehension of texts. The DHH children with syntactic deficit made significantly more errors in reading aloud and more comprehension errors than the DHH children with intact syntax (and than the hearing controls), even though most of them did not have dyslexia at the word level. The DHH children with syntactic deficit made significantly more reading errors when they read texts with syntactic movement than on matched texts without movement. These results indicate that difficulties in text reading, manifesting both in errors in reading aloud and in impaired comprehension, may stem from a syntactic deficit and may occur even when reading at the word level is completely intact.

## 1. Introduction

To read a text correctly, the reader needs both word-level reading abilities as well as syntactic and lexical abilities [1]. For example, to read aloud the sentence “The researcher who read the paper smiled”, with the phrasing intonation in the correct places, one needs to construct correctly the syntactic structure of this sentence. Similarly, to read the word “presents” in “The paper presents evidence for the effect of syntax on reading”, one needs to analyze the syntactic structure and understand that presents is the main verb, and hence read it as a verb, with final stress, rather than as a noun with stress on the first syllable. Like oral reading, the comprehension of written texts is also dependent on word-level reading abilities as well as linguistic abilities. Looking through this window of the multiple abilities required for text reading, we examine the difficulties of some children who are deaf or hard of hearing (DHH) in text reading and text comprehension.

Pioneering studies of text reading of DHH children reported severe difficulties [2,3,4,5,6]. Recent years saw technological developments in hearing aids and cochlear implants, increased newborn hearing screening, and growing use of cochlear implants, all of which improve certain aspects of spoken language of DHH children. Nevertheless, studies still show that alongside DHH children whose text reading is similar to that of hearing controls, other DHH children find it difficult to understand texts [7,8,9,10,11,12].

Some researchers ascribe the text comprehension difficulties displayed by DHH to impaired decoding at the word level, which, in turn, stems, according to these researchers, from poor phonological processing and poor phonological awareness [13,14,15,16,17,18]. Growing body of evidence, however, shows that many DHH children do not experience word-level decoding difficulties but still show difficulty in reading comprehension [12,19,20]. Ref. [5,19,21] claim that word-level reading deficits are not the source of their difficulties in reading texts, but rather language deficits; Indeed, researchers ascribe the difficulty in reading comprehension to the linguistic knowledge needed to process words at the sentence or paragraph level: limited vocabulary, poor morphological knowledge, and poor syntactic abilities lead to the failure to extract meaning from the written text [22,23,24,25].

Here, we examine the possibility that syntactic deficits underlie the difficulty that some DHH children have in reading aloud of text and in text comprehension and that what distinguishes children with and without text reading difficulties is their syntactic abilities at the sentence level. We also examine the abilities of these children in reading at the single word (and nonword) level.

### Syntactic Impairment Displayed by DHH individuals

At the sentence level, many DHH children encounter difficulties in understanding non-canonical sentences that are derived by syntactic movement [26,27,28,29,30,31,32,33]. Studies that assessed the syntactic abilities of DHH children in various languages found difficulties in the comprehension and production of object relative clauses (English: [26,34,35,36]; Hebrew: [30,31,32,37,38]; Arabic: [32,39,40]; Italian: [41,42]; German: [43]), in the comprehension and production of object questions (English: [28,44]; Hebrew: [31,33,45,46]; Standard Arabic and Palestinian Arabic: [32,47]; German: [43]; Italian: [42,48]), and in the comprehension of topicalization structures (Hebrew: [30]; Arabic: [39,40]; German: [43]).

These three impaired syntactic structures—object relative clauses, object questions, and topicalization structures—share a common syntactic characteristic: They are all derived by movement of a phrase across another phrase (the object moves across the subject), to the beginning of the clause. This specific type of movement, of a phrase to the beginning of the sentence, is called “Wh-movement” (or “A-bar movement”), because it is the movement that derives Wh-question. As shown in examples (1)–(3), this Wh-movement, depicted by an arrow in these examples, in which a phrase leaves its original position (marked with an underline) and is pronounced in an earlier position in the sentence, creates a non-canonical order of the arguments in the sentence, where the agent of the action follows, rather than precedes, its theme.


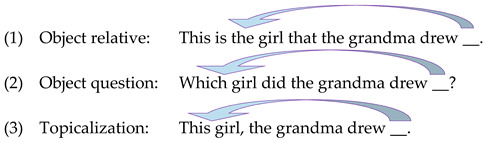


In addition to Wh-movement difficulties, some DHH children were reported to have difficulties also in structures with verb movement to the second position in the sentence, before the subject (also termed “V-to-C movement” or “triggered inversion” [49]. Below, we refer to this movement for simplicity as “verb movement”). In English, this type of verb movement can be seen only in the movement of the auxiliary verb in questions to a position before the subject, as in “have you seen the moon tonight?”. Such movement was found impaired in English-speaking DHH individuals [34,44]. In other languages, such as Hebrew and German, verb movement applies not only to auxiliary verbs and not only to questions, but to all kinds of verbs, as in example (4) translated word-by-word from Hebrew. In Hebrew, movement of the verb to the second position in the sentence, before the subject, is optional, so that both “Yesterday ate the girl hummus” and “Yesterday the girl ate hummus” are acceptable [49,50,51]. Difficulties of DHH in such verb movement structures were reported for Hebrew [52,53,54] and German [43].


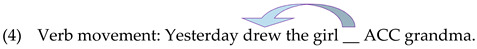


This syntactic movement deficit has been ascribed to the insufficient language input during the critical period for the acquisition of syntax of a first language, which is probably the first year of life [30,31,46,55,56,57,58]. That is to say, most DHH children who display syntactic difficulties are not born with a syntactic deficit, but this is rather a result of insufficient language input. Many DHH children who are born to hearing parents and communicate using oral language do not get enough language input during the first year of life and until they are fitted with effective hearing devices. This also accounts for the consistent finding that some DHH children show intact syntactic abilities whereas others show impairment in syntactic movement. When examining the age of exposure to language, the DHH children with impairment in syntactic movement are in most cases the ones who were born DHH but received hearing aids or cochlear implants late, after they were one year old. DHH individuals who received sufficient language input during the first year of their lives, thanks to early use of hearing devices, often show age-appropriate comprehension and production of sentences with syntactic movement [30,31,46].

The fact that some DHH children have intact syntactic abilities whereas other DHH children display syntactic impairment would allow us to examine the effect of syntax on text reading and comprehension, by comparing the text reading of the two groups.

In the present study we explore our main research question as to whether specific syntactic deficits underlie the difficulties of DHH children in text reading, both reading aloud and comprehension. We examine this in two main ways. We compare text reading of DHH children with and without syntactic impairment (assigned to the two groups on the basis of extensive syntactic testing), and we also compare their reading of paragraphs that include sentences with syntactic (Wh- and verb-) movement to their reading of paragraphs without movement.

If indeed the syntactic deficit in movement structures, combined with the abundance of these structures in texts, are responsible for DHH children’s difficulties in text reading and comprehension, we expect DHH children with syntactic difficulties to show poorer text reading than DHH children without syntactic difficulties. We also expect, for DHH children with syntactic impairment, more errors in reading and comprehension of texts with the relevant movement structures than texts without such structures.

We describe below the assessment of the syntactic abilities of the DHH participants and the assessment of their reading aloud at the single word level, according to which we determined for each participant whether they have a syntactic deficit in syntactic (Wh- and verb-) movement and whether they have a deficit in reading aloud at the single word level. We then move to the main experiment of this study, assessing their text reading and text comprehension in paragraphs with and without syntactic movement, and their relation to their syntactic and word reading abilities.

## 2. Participants

The DHH participants were 32 Hebrew-speaking children. They were 16 boys and 16 girls, aged 9;1–12;2 years (M = 10;6, SD = 0;9). They had moderate to severe hearing loss from birth and use spoken language (Table 1).

At the time of testing, all the participants were studying in primary schools in hearing classes with inclusive schooling using oral education, and each of them received additional support from a special teacher for DHH children, 2–4 h a week. All the participants consistently wore binaural hearing aids (15 children) or used cochlear implants (17 children, 4 of them used two cochlear implants). The background information on the participants’ hearing is presented on Table 1. An informed consent statement approved by the Ministry of Education Review Board (Ethics approval 9327/639) was signed by the parents of all participants; the study was also approved by the Tel Aviv University ethics committee (Ethics approval 132.14).

All the participants passed a hearing screening test, performed while they were wearing their hearing aids/ implants, in which they were asked to repeat 10 sentences that included sibilants, that the experimenter read to them with her lips concealed.

The control participants for the text reading experiment were 18 hearing children aged 8;10–10;7 (M = 9;11, SD = 0;5) in 4th–5th grade, who were matched in age and grade to the youngest children in the DHH group. They were 14 girls and 4 boys, all typically hearing native speakers of Hebrew, with typical language development. (One of the hearing control participants read only 4 of the 6 syntactically complex texts). The control groups of each of the background syntactic tests are described in Table A1 in Appendix A.

## 3. Assessment of Syntactic Abilities

To establish which of the DHH children had a syntactic deficit in movement structures, we assessed the syntactic abilities of each DHH participant using six syntactic tests, mainly focusing on Wh-movement. Three tests assessed sentence comprehension of relative clauses and Wh-questions (auditory comprehension of subject and object relative clauses –sentence-picture matching task with 40 items; auditory comprehension of relative clauses and Wh-questions—a picture selection task with 80 items; comprehension of written object relatives: paraphrasing of object relatives with center embedding with 20 items); 2 tests assessed the production of subject and object relative clauses (Elicitation of relative clauses using a picture description task with 20 items, and Elicitation of relative clauses using a preference task with 20 items), and one test assessed repetition of sentences derived by Wh-movement and of sentences with verb movement (70 items). The description of the syntactic tests and their results at the group and individual levels are given in Appendix A.

According to the performance of each DHH participant in these 6 syntactic tests, we determined whether they had a syntactic impairment in Wh-movement or not. We extracted 8 litmus measures from the 6 tests that indicate Wh-movement abilities, as shown in Table 2: 4 measures on the comprehension of object relatives and object questions, from the three comprehension tasks, 3 measures on the production of subject- and object relatives from the two production tasks, and a combined measure of repetition of structures derived by object Wh-movement: object relatives, object questions, and topicalizations. Performance that was significantly below that of the hearing controls in each task was determined using Crawford and Howell’s [59,60] *t*-test for the comparison of a single subject to a group, with an alpha level of 0.05.

We included a DHH child in the syntactic impairment group (hence: LOWSYD, LOW Syntax DHH—DHH children whose syntactic abilities are lower than typically-hearing children their age) when s/he showed performance that was significantly below the hearing controls in object Wh-movement in at least three of these measures, and failed in at least one comprehension task and one production task. 

Children who showed performance that was within the range for typically-hearing children their age in all six tasks or who showed below-control performance in only one measure were included in the good-syntax group (GOODSYD, GOOD Syntax DHH–DHH children whose performance in the syntactic tests was within the range for typically-hearing children their age).

According to these criteria, we included 14 DHH children in the syntactic impairment (LOWSYD) group (the first 14 participants in Table 2); 10 of them also showed a deficit in repeating sentences with verb movement. Fifteen DHH children were considered unimpaired (GOODSYD), as they performed in all or most of the tests like the hearing controls (the last 15 participants in Table 2).

Three DHH children were excluded because they showed a borderline impairment or an impairment that was not a classic Wh-movement impairment and therefore were not included in further analyses (see Table 2. YIBM showed impairments in the syntactic and word reading tests that were not Wh-movement based: he showed a deficit characteristic of a phonological output buffer deficit [61,62]), which resulted in problems in head movement (verb movement and construct state nominals and in overuse of resumptive pronouns), and in function words and morphological substitutions. LILF showed impairment only in (some of) the comprehension tasks; SHVM showed impairment only in one comprehension task and showed marginal impairment in one production task. These participants were therefore excluded from further analyses). Table 2 summarizes the performance of each of participants in comparison with the control group on each of the tests and litmus measures.

## 4. Assessment of Reading at the Word Level

The main goal of this study was to understand the bases of the difficulties DHH children experience in text reading. We suggested that a crucial basis for these difficulties could be a syntactic impairment and that structures derived from syntactic movements in a text affect reading and reading comprehension at text level. However, it is also possible that the difficulties result from a deficit in reading already at the word level. We therefore examined the participants’ reading at the word level.

### 4.1. Method: “TILTAN”-Oral Reading Screening Test

The ability of each participant to read aloud at the word level was tested using a reading screening test—TILTAN [63]. The TILTAN test includes reading aloud of a word list (136 single Hebrew words, 2–11 letters long); a nonword list (30 nonwords: 20 with and 10 without diacritics, 3–6 letters long), and a word pair list (30 word pairs, 3–6 letters long). The TILTAN has a reliability coefficient of 0.968 [64] based on a sample of 1022 Hebrew-readers with and without dyslexia [65].

The screening test includes words and nonwords of various types that can reveal impairments in the various components of the word reading process, i.e., the various types of dyslexia (For information on the various types of dyslexia, see [66]). Appendix B details the properties of the various items in the TILTAN.

The participants read the TILTAN lists aloud from a booklet of A4 pages. In line with the TILTAN manual, if the participant made an error on an item, even if they later corrected it, we analyzed the error. The test was audio-recorded, and the experimenter wrote the participant’s responses during the test and checked and corrected the scoring using the recording.

On the basis of this test, we determined whether a participant had intact reading or whether s/he had dyslexia, and if s/he had dyslexia, which types of dyslexia were suspected based on the error pattern s/he showed and the factors that affected her/his reading (frequency effect, word length effect, lexicality effect, etc.) Impaired performance in the screening task was determined according to the comparison of the participant’s reading to an age-matched control group (20 typically-developing hearing Hebrew-speaking children in fourth and fifth grade, mean age = 10;5, SD = 0;9), using the Crawford and Howell’s [60] *t*-test for the comparison of an individual to a control group. Impaired performance was defined as performance that was significantly below the control, with *p* < 0.05. The type of dyslexia was determined using the same procedure and statistical test, applied to the various types of errors, i.e., we determined that a participant had a certain dyslexia if they made significantly more errors of the relevant type compared to the control group (e.g., we determined that a participant had letter position dyslexia if they made significantly more letter position errors than the controls).

### 4.2. Results

The reading screening test, which is very sensitive to reading difficulties, showed that most of the DHH children had intact reading at the word (and nonword) level: 22 DHH participants had good word-level reading, which was age-appropriate, and 7 children had an impairment in reading at the word level (3 additional participants, who were borderline in the syntactic tasks, were not included in further analyses including the word level reading). For more information on the results of the word reading screening test, see Appendix B. Of the seven children with the reading impairment, four children (DORF, HIMF, LIHF, and RARM) showed regularization errors, which are characteristic of reading via grapheme-to-phoneme conversion (e.g., reading “listen” with a pronounced “t”, “have” as rhyming with “gave”, or pronouncing “now” as “no”). Namely, these 4 DHH children often did not read via the lexical route and instead read words via the sublexical route, as new words (see [66] for a detailed reading model). It may very well be that this reading pattern is not an indication of dyslexia but rather of insufficient lexical exposure leading to only partial phonological lexicon. The vocabulary of DHH children is often less complete than that of hearing children [67,68,69]. When a lexical entry is not present in the phonological output lexicon, reading cannot take place via the lexical route, and as a result, the child needs to read via the sublexical route, yielding slow and inaccurate reading, especially of irregular words [66,70,71,72,73].

Only 4 of the 29 tested DHH participants showed dyslexia (beyond the lack of lexical entries leading to sublexical reading). All four had dyslexias that resulted from deficits in the early stage of reading—the orthographic-visual analysis stage. This stage is responsible for letter identification, letter position encoding, and letter-to-word binding. The reading test indicated that participants YAZM and AVCM had letter position dyslexia and attentional dyslexia, which cause letter migrations within and between words, respectively [74,75,76,77,78], as well as vowel dyslexia, which causes errors in reading vowel letters [79]; Participants LIHF and CHBM had a more generalized deficit in the orthographic input buffer, resulting in letter migrations within and between words as well as letter omissions and substitutions in long words and nonwords, and morphological errors in reading [66,80]; LIHF also had surface dyslexia.

## 5. Reading Aloud and Comprehension of Paragraphs with or without Syntactic Complexity

We examined the effect of syntactic complexity and syntactic impairment on reading aloud and on reading comprehension using short paragraphs that included sentences with syntactic movement. We compared reading and comprehension in these paragraphs to matched paragraphs with only simple sentences, similar in length, words, and content. We also compared the performance of DHH participants with and without syntactic deficits.

We included in the paragraphs with syntactic movement sentences with Wh-movement and verb movement. Both Wh-movement and verb movement are commonly used in written Hebrew texts. In a count of 6074 sentences in children’s books and second grade textbooks, we found that a third of the sentences (34%) are derived by Wh-movement, and 19.4% of the sentences included verb movement to second position [81].

### 5.1. Materials

The test included 12 short paragraphs (65–105 words long each). Six of the paragraphs included sentences derived by Wh and verb movement. Each of the other six paragraphs were matched to one paragraph with movement in length, in content, and in the words used but included only simple sentences, without these syntactic movements. Therefore, the only difference between each two matched paragraphs was whether or not they included movement. For example, if the movement paragraph included a sentence with verb movement like “one day went the children to the zoo”, the matched paragraph included the parallel sentence without verb movement “last year the kids went to the zoo”. When the movement paragraph included a sentence with Wh-movement like the object relative “The hikers that the monkeys scared ran away”, the matched paragraph included the parallel sentence without Wh-movement “The monkeys scared the hikers and the hikers ran away”. Table 3 presents an example of matched paragraphs. 

The sentences with Wh-movement (and their matched simple sentences) were semantically reversible so each of the noun phrases in the sentence could be the agent or the theme of the action. In this way, the meaning of these sentences could not be determined without syntax, solely on the basis of lexical and world knowledge. The two noun phrases in each sentence were of the same gender and number so that the verb inflection, which agrees in Hebrew with the subject, could not be used to identify the agent.

The paragraphs with movement included a total of 56 sentences. A total of 48 sentences of which included movement (14 sentences included more than one type of movement). These included 35 sentences with Wh-movement: 25 object relative clauses with final or center embedding, 2 PP object relatives, 2 subject relative clauses with left or center embedding, and 6 object topicalization; and 21 sentences with verb movement. Six of these movement-derived sentences also included embedding of a sentence to a verb. Four additional sentences were simple sentences without Wh and verb movement and four were embedded sentences with embedding and without Wh and verb movement. (The paragraphs without syntactic movements did include construct state nominals, which are very common in Hebrew and do include another type of movement (N-to-D movement). The DHH participants showed a remarkable difficulty in these structures. Because construct state nominals are structures with movement, in our analyses of errors in the texts without movement we do not include errors in these structures. We will not discuss these here; for detailed analysis of the DHH children’s reading of these nominals, see [82]. (See Appendix C for a detailed list and examples of the various structures in the paragraphs.)

### 5.2. Procedure

Each of the 12 paragraphs was printed on an A5-sized card. The participant received a card and was asked to read the paragraph aloud. After the participant completed reading the paragraph, the experimenter asked 4–5 comprehension questions, with a total of 56 comprehension questions—28 for the paragraphs with syntactic movement and 28 for paragraphs without syntactic movement. All the questions asked about information that explicitly appeared in the text. For instance, questions that were presented with the paragraphs in Table 3 were the following: What did the travelers see? Who surprised whom? Who threw stones? Who ran behind a hill? What did the monkeys do after the travelers ran away? These questions referred to sentences derived by Wh and verb movement (and, in the matched control paragraphs, to the matched sentences) and referred to the object and/or subject of these sentences. Object questions (e.g., Whom did the monkeys surprise?) are hard to comprehend for DHH children [31], therefore, when an object question was presented and the participant did not answer it, the experimenter rephrased the question (e.g., Who surprised whom?). The questions for each pair of matching paragraphs with and without movement were identical in structure. The number of comprehension questions of each type is presented in Appendix C, Table A4. The participants’ reading aloud and their answers to the questions were audio-recorded. No time limit was set. To reduce demands on memory, the paragraphs remained in front of the participants throughout the reading and while answering the questions. The participants were encouraged to look for the answers in the paragraphs. If the answer given by the participant was not clear to the experimenter, she asked the participant a clarification question.

The 12 paragraphs were divided into two blocks. Each block was administered in a separate session. In the first session, the participants read 3 paragraphs with syntactic movement and 3 paragraphs without. In the second session, the participants read the 6 parallel paragraphs, 3 paragraphs with syntactic movement and 3 paragraphs without. The two parts of the test were administered one or two weeks apart.

### 5.3. Results: Errors in Reading Aloud of Paragraphs with Movement

#### 5.3.1. Reading at the Single Word Level Does Not Explain Reading Errors in Text

In Section 4, we assessed the reading at the word level of each DHH participant. This now allows us to examine whether their difficulty in text reading resulted from a deficit in reading at the word level. The results were clear-cut: although most of the DHH participants (22 of the 29 participants) did not have reading difficulty at the word level, they made many reading errors while reading the paragraphs with syntactic movements. Of the 16 children who had more errors in reading the texts aloud than the hearing controls, 9 had no reading difficulty at the single word level. Moreover, even for the children who did have dyslexia, the types of errors they made in reading the texts aloud could not be explained by their dyslexia: For example, omission of words that are object case markers cannot be accounted for by letter position dyslexia, attentional dyslexia, vowel letter dyslexia, or surface dyslexia; neither can these dyslexias explain word order errors or morphological errors that add or omit a multi-letter affix. Furthermore, as we will show below, a deficit in reading at the word level would not be able to account for the significant difference in reading error rates between the paragraphs with and without movement.

#### 5.3.2. Errors in Reading Texts with Movement of DHH Children with Syntactic Impairment Compared with DHH Children with Intact Syntax

To examine whether syntactic difficulties lead to DHH children’s text reading errors, we compared the accuracy in reading aloud of the paragraphs with movement of the two DHH sub-groups—those with impaired syntax (LOWSYD) and those whose syntax was intact (GOODSYD). The two DHH groups were compared to each other and to the hearing control group.

**Figure 1:**
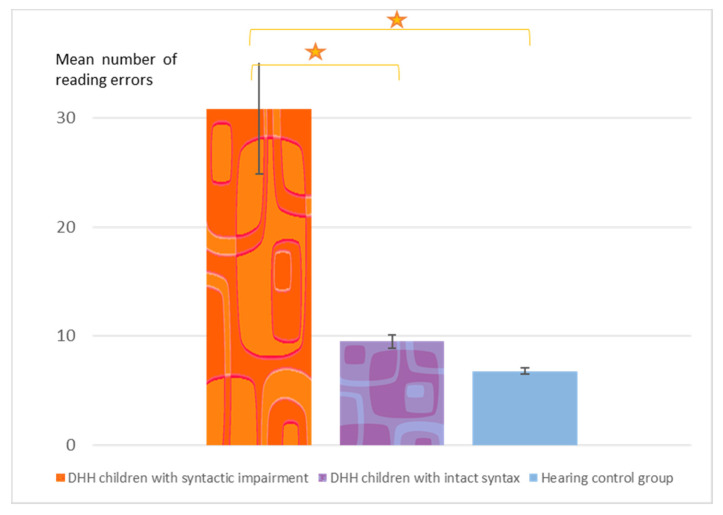
Mean number of reading errors (and SD bars) in paragraphs with syntactic movement in the three groups (out of 56 sentences, 48 of which included movement). Star: *p* < 0.0001.

The rate of reading errors in each group is presented in Figure 1. In reading aloud the 56 sentences in the paragraphs with movement, the LOWSYD made an average of 30.8 errors (SD = 19.3), three times more reading errors than the GOODSYD, who read the same paragraphs with only 9.5 errors on average (SD = 6.3). The groups differed significantly in their accuracy in reading aloud of the texts with movement, Welch’s F(2, 23.53) = 10.36, *p* = 0.001. A Games-Howell post hoc analysis (used since the homogeneity of variance assumption was not met) indicated that the LOWSYD made significantly more errors in reading aloud than GOODSYD (Mean difference = 21.32, *p* < 0.0001) and than the hearing controls (who made 6.8 errors, SD = 4.6, Mean difference = 23.96, *p* < 0.0001). The GOODSYD group did not differ from the hearing controls (Mean difference = 2.64, *p* = 0.39). (Even when we include in the LOWSYD-GOODSYD comparison only the DHH participants who had a syntactic deficit and no deficit in reading at the word level, the two groups still differ significantly, t(20) = 3.94, *p* < 0.0004, d = 1.89).

This large difference in the number of reading errors in the texts with syntactic movement between the DHH participants with and without syntactic impairment suggests that what underlies the reading difficulty some DHH children experience is their syntactic impairment rather than their hearing loss.

#### 5.3.3. Types of Reading Errors in Paragraphs with Syntactic Movement

##### Types of Reading Errors in the Different Groups

The reading errors of the LOWSYD children often formed ungrammatical sentences. We classified the reading errors into the following categories: structural errors (word order errors, structure changing errors, reading verbs as nouns and nouns as verbs, omissions and substitutions of embedding markers, omission and substitutions of object case markers, and incorrect intonation-phrasing that indicate a lack of understanding of the structure of the sentence), lexical errors (additions, omissions, and substitutions of words) that do not affect the syntactic structure of the sentence, errors in function words, and errors related to the definite determiner. Whereas the structural errors occurred almost exclusively in the texts with syntactic movement, the lexical errors, errors in function words, and errors in determiners occurred in both types of text; we will not discuss these types of errors here. The frequency of the various error types made by the three groups is presented in Table 4.

##### Structural Errors in Reading the Various Target Sentence Structures

*Reading direct object relatives.* The main error types that the LOWSYD children made in reading object relatives were omissions of the obligatory relativizer “that” (21 errors), incorrect reading of the verb or its omission (22 errors), incorrect reading of the subject—sometimes reading the subject noun phrase as a verb, or omitting it altogether (16 errors), addition of a coordinator before the verb of the main clause (6 errors, e.g., reading “The big bear that the elephant sprayed was very angry” as “The big bear that the elephant sprayed and was very angry”), and intonation (sentence phrasing) errors that reflect misunderstanding of the structure of the sentence (25 errors). See Appendix D for examples for various types of errors in reading aloud.

All these types of errors indicate a failure to comprehend the syntactic structure of object relatives. The omission of the relativizer and the addition of the coordinator between the verb in the relative clause and the main verb are evidence of the difficulty to comprehend the subordinate structure of the relative clause. The erroneous reading of the verbs and the nouns is evidence of the difficulty in identifying the structure of the sentence, its verb, and its arguments.

*Reading Sentences with topicalization.* The main error types that the LOWSYD children made while reading topicalization sentences were omission of the object case marker “et” preceding the definite object (5 errors) and addition of an object case marker before the definite subject (3 errors) (see examples in Appendix D). These errors created sentences with two subjects and no object or with two objects and no subject. Such errors indicate difficulty in assigning thematic roles to arguments within topicalization sentences. Importantly, these errors did not result from a difficulty in case markers: When these children read the six simple sentences parallel to the topicalization sentences (in the parallel paragraphs without the movements) they never omitted or added object case markers - none of the case marker errors in the simple sentences changed the object to a subject or vice versa.

*Reading sentences with verb movement.* In reading sentences with verb movement, the LOWSYD children mainly read the verb incorrectly (10 errors), omitted the subject (2 errors), added an object case marker to the subject (4 errors), omitted a determiner from the subject in a way that makes it the object (2 errors), and read the subject as the object (2 errors). All these types of errors indicate a difficulty to comprehend the syntactic structure of sentences with verb movement. The children find it difficult to assign the thematic roles to the arguments in the sentence when the verb has lost its original position between the subject and the object. Because the subject follows the verb in verb movement sentences, rather than precedes it, they often took it to be the object and therefore the theme, rather than the agent, of the action.

*Reading sentences with sentential complements of verbs.* Some of the LOWSYD also made errors in reading sentences with sentential complements of verb. In 10 cases, they omitted the embedding marker “she-”, which is obligatory in Hebrew (whereas the GOODSYD children only omitted it twice). Such ungrammatical omissions are evidence of a difficulty in embedding some of the LOWSYD had even when the sentences have no movement.

#### 5.3.4. Self-Corrections

**Figure 2:**
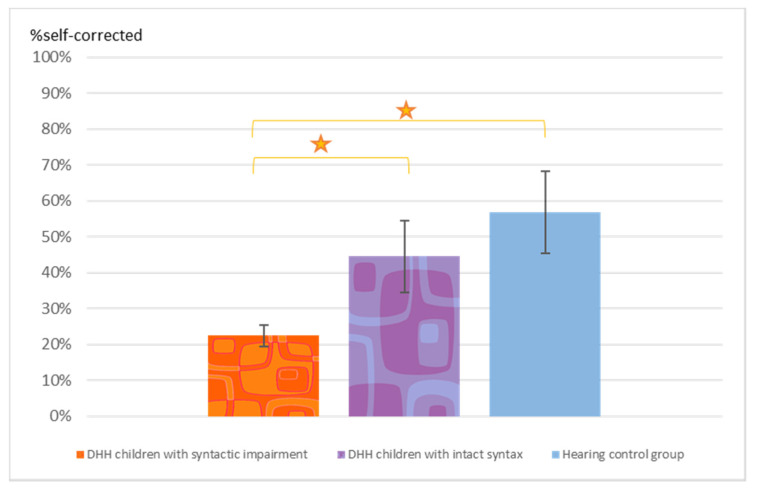
Percentage self-corrections of reading errors in the texts with movement. Star: *p* < 0.0001.

Another measure that showed important differences between the LOWSYD and GOODSYD groups was the rate of self-corrections they made while reading the paragraphs with syntactic movement. Whereas in the hearing control group and in the GOODSYD group about half of the reading errors were immediately self-corrected (56.8% and 44.5%, respectively), the LOWSYD group corrected only one fifth of their reading errors (22.4%), as shown in Figure 2.

Namely, whereas the children who did not have syntactic deficits—the hearing controls and the GOODSYD—noticed they made a reading error, stopped and corrected themselves in about half of the cases, the LOWSYD did not notice their errors or were unable to correct them, so they corrected only very few of their errors. The groups differed significantly in their tendency to self-correct, Welch’s F(2, 26.62) = 17.68, *p* < 0.001. A Games-Howell post hoc analysis (used since the homogeneity of variance assumption was not met) indicated that the LOWSYD corrected themselves significantly less often than the GOODSYD (Mean difference = 23.30, *p* < 0.0001) and than the hearing controls (Mean difference = 34.34, *p* < 0.0001). The GOODSYD did not differ in the rate of self-corrections from the hearing controls (Mean difference = 11.03, *p* = 0.34).

### 5.4. A Comparison between Paragraphs with and without Syntactic Movement

The LOWSYD children, who made many reading errors when they read aloud the paragraphs with syntactic movement, made significantly fewer errors when they read the parallel paragraphs without syntactic movements, t(13) = 5.52, *p* < 0.0005, d = 0.9. Table 5 summarizes the types of errors the LOWSYD made in the two kinds of paragraph. The few errors the LOWSYD children made when reading the paragraphs without syntactic movement were mainly related to the definite determiner (42.2%) and to function words (18.6%). (They also made errors in construct state nominals, but as we reported above and discussed in [82], construct state nominals are structures with movement.) The pattern of more reading errors in the paragraphs with syntactic movement than in the paragraphs without movement held for each of the individual participants in the LOWSYD group, including the dyslexic participants, and was significant for each of them except two (who made more errors on the paragraphs with movement than on the paragraphs without, but not significantly so, one was dyslexic, the other with typical reading at the word level).

**Figure 3:**
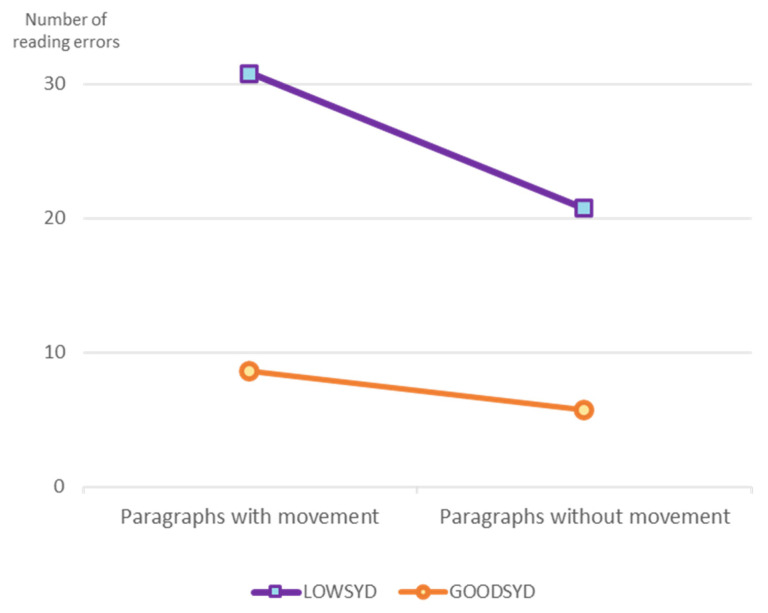
Reading errors: The interaction between group of DHH children, with or without syntactic impairment, and type of paragraph, with or without syntactic movement.

The GOODSYD children did not show this difference between reading errors in paragraphs with movement (9.4%) and paragraphs without movement (5.7%), as shown in Figure 3. The interaction between groups (LOWSYD, GOODSYD) and paragraph type (with and without movement) was significant, F(1, 26) = 6.33, *p* = 0.02. 

### 5.5. Comprehension Questions on Texts with Movement

**Figure 4:**
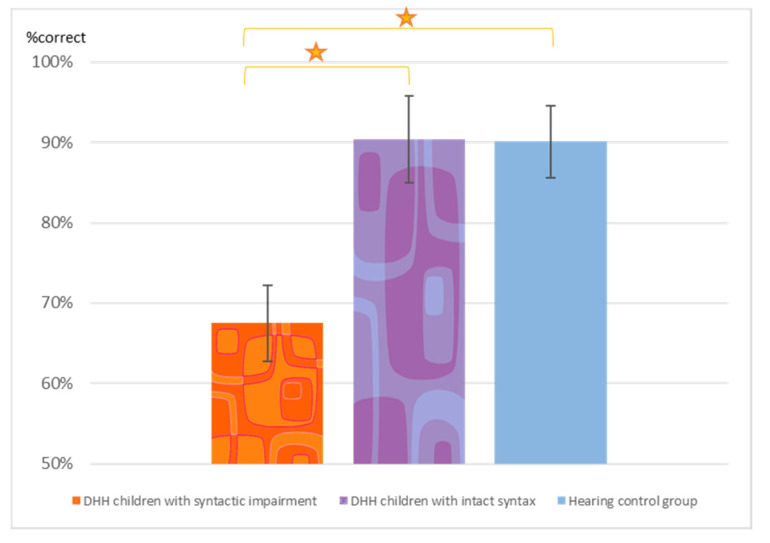
Percentage correct answers to the comprehension questions about the texts with movement. Star: *p* < 0.0001.

The LOWSYD showed difficulties not only in reading the texts with syntactic movement aloud but also in answering the comprehension questions about these texts, as summarized in Figure 4. They provided correct answers to only 67.5% of the questions posed to them (SD = 7.3%), whereas the GOODSYD answered the comprehension questions far better (M = 90.4%, SD = 5.9%). The groups differed significantly in their ability to answer the comprehension questions, Welch’s F(2, 24.52) = 18.26, *p* < 0.001. Games-Howell post hoc procedure (used since the homogeneity of variance assumption was not met) indicated that the LOWSYD made significantly more errors in answering the comprehension questions than the GOODSYD (Mean difference = 6.40, *p* < 0.0001) and than the hearing controls (Mean difference = 6.30, *p* < 0.0001). The GOODSYD group did not differ from the hearing controls (Mean difference = 0.098, *p* = 0.99).

Eleven of the 14 LOWSYD children performed significantly below the hearing control group in answering the comprehension question on the paragraphs with the syntactic movement (i.e., these 11 children provided 14–21 correct answers out of the 28 questions). In contrast, only one of the 15 GOODSYD children showed performance below the hearing controls on the comprehension questions, answering 22 questions correctly.

The LOWSYD participants produced three types of errors in their answers to the comprehension questions. The most common type of error (82%) was providing an incorrect noun phrase in response to the question, 96% of these errors (99 of 103) were selecting the other noun phrase that was mentioned in the sentence, indicating the failure to comprehend the thematic roles in the sentences they read (Example 5). Other 14% of the answers were cases in which the child read aloud a whole sentence from the text but was unable to extract the relevant answer to the specific question from it (Example 6). Finally, 5% of the incorrect answers were partial answers to a question that required two items of information from the paragraph.

(5) Selecting an incorrect noun phrase from the same sentence.
  The sentence in the paragraph to which the question refers: The monkeys that the travelers expelled threw stones.
  The question: Who threw stones?
  The answer: The travelers threw stones.

(6) Quoting a sentence (or part of it) without extracting the relevant answer.
  The sentence in the paragraph to which the question refers: The monkeys that ate everything that the travelers left cheered.
  The question: What did the monkeys do after the travelers ran away?
  The answer: Everything that the travelers left cheered.

## 6. Discussion

Many studies report that DHH children have reading difficulties [2,3,4,8,83]. In this study, we asked what exactly “reading difficulties” means. We distinguished between reading at the word level, reading aloud of texts, and the comprehension of written texts. We separately assessed their abilities at these different levels. We assessed their reading at the word level using a dyslexia test that assessed their reading aloud of lists of words and nonwords; we tested their reading aloud of 12 texts, and we tested their text comprehension using comprehension questions on the texts they had read.

These assessments indicated that most of the DHH participants did not have dyslexia, and their word and nonword reading was similar to that of hearing children their age. Many of them did, however, show a considerable difficulty in reading specific types of texts aloud and in understanding them.

### 6.1. A Strong Relation Between Syntactic Deficit and Errors in Reading Texts Aloud

To examine the hypothesis that the difficulty children with DHH have in reading aloud of texts stems from a deficit in syntax, we separately assessed their syntactic abilities.

We tested their comprehension and production of sentences with syntactic movement (relative clauses, wh-questions, topicalized sentences, and verb movement structures) using 6 syntactic tests. According to their performance in these syntactic tests, we determined, for each of the 32 DHH children whether they had a syntactic deficit or not. This analysis yielded a group of 14 DHH children with a syntactic deficit and 15 DHH children with intact syntax (3 additional children had a marginal deficit and were excluded from further analyses). We compared the way these two subgroups read texts aloud and compared their reading aloud of texts with and without syntactic movement.

The results showed a tight connection between the syntactic deficit that many DHH children have, and their text reading difficulties. The DHH children whose syntax was impaired made far more errors in reading aloud of texts with syntactic movement than the DHH children whose syntax was intact. They made far more errors in texts with syntactic movement than in parallel texts that did not involve sentences with syntactic movement. These findings suggest that the reading difficulties of DHH children stem from their syntactic deficit.

Many studies reported that orally-trained DHH children have difficulties in the comprehension and production of structure derived by syntactic movement, such as object relative clauses, object Wh-questions, and topicalized structures [26,27,28,29,30,31,32,33,34,35,36,37,38,39,40,41,42,43,44,45,46,47,48]. These structures are derived by movement of the object noun phrase across a subject noun phrase. The current results indicate that this deficit has implications for text reading as well, hampering the reading aloud (and comprehension) of texts that include such sentences.

This was also evident in the types of errors they made in reading the texts aloud. They made errors of changing the word order, verb omissions, object case errors, reading nouns as verbs and verbs as nouns, omissions of relativizers (embedding markers) in relative clauses and of other function words, and more. These errors indicated that they did not understand the thematic structure of the sentences with movement, namely, they did not understand who did what to whom in these sentences. Such errors are also a further indication that their difficulties did not result from dyslexia (a reading deficit at the word level), because no dyslexia yields such pattern of reading errors.

The syntactic deficit also affected their ability to correct themselves once they made a reading error in the texts: Unlike the DHH children whose syntax was unimpaired (and unlike the hearing controls), who self-corrected about half of their reading errors, the DHH children whose syntax was impaired rarely tried to correct themselves. Namely, even though most of their reading errors created ungrammatical and uninterpretable sentences, they either did not notice that or could not correct the sentences, as they did not understand the written sentence.

These results indicate that DHH children make errors in reading aloud if they have a syntactic deficit, when they are required to read a sentence that involves a syntactic structure that is difficult for them to comprehend. They did not have a general deficit in reading aloud, as indicated both by the findings of a word-level reading task, in which most of them showed age-appropriate performance, and by the fact that they read texts without syntactic movement with far fewer reading errors. Namely, their reading errors stemmed not from a decoding difficulty but rather from a syntactic difficulty. Because they could not understand the sentences, they could not read them correctly. Barajas et al. [19] found similar results with respect to text comprehension: when they tested how decoding abilities and the comprehension of various complex sentences (with respect to movement-derived sentences they tested 4 passive sentences) explain reading comprehension in deaf Spanish elementary school students. They found that most of their participants (43 of 47) performed well on tasks of reading aloud of single words but still more than half of them (26) showed difficulties in text comprehension. Their comprehension of syntactically complex sentences explained a large part of the variation in text reading. This led Barajas et al. to conclude, similar to our conclusions here (and see the next Section 6.2), that the reading comprehension problem of deaf students does not stem from an inability to translate the written words to their phonological representation and that it is related to their syntactic abilities.

### 6.2. The Syntactic Deficit Causes Difficulties in Text Comprehension

The results also show that the syntactic deficit affected the comprehension of texts, as indicated by their performance in answering comprehension questions. The DHH children with syntactic deficit had significantly more difficulties understanding the texts with syntactic movement than the DHH children without syntactic deficit.

The effect of the syntactic deficit on the comprehension of the texts is hardly surprising, as we would expect children who have difficulties understanding certain types of sentences to also have difficulties understanding these sentences in written texts. However, this finding stresses that even though the texts provide a context, which could have assisted in the comprehension of sentences with movement, the children still struggled with understanding them and could not use the context to answer the questions correctly.

### 6.3. DHH Children’s Reading at the Single Word Level

#### 6.3.1. Errors in Text Reading Do Not Result from Dyslexia

We thoroughly assessed the DHH participants’ reading at the word and nonword level using a dyslexia screening test that is sensitive to the various types of dyslexia. Still, almost all of the DHH participants showed typical reading for their age at the word and nonword level. Of the 29 children, 22 had age-appropriate reading, and only 4 had some sort of dyslexia (and 3 additional participants read via the sublexical route, see Section 6.3.2).

These results suggest that the text reading difficulties of most of the syntactically impaired DHH children did not stem from dyslexia. An additional support for this conclusion comes from the finding that these participants made far fewer errors when they read the texts without movement. Had their deficit been a result of a deficit at the word level, they should have made reading errors on the texts without movement as well. 

Additionally, most of the types of errors the DHH children made in text reading are not characteristic of any kind of dyslexia, as no dyslexia is expected to cause word order errors, verb omissions, or misplacement of object case markers. (Deep dyslexia is a type of dyslexia that may cause difficulties in reading verbs and function words [66,84,85], but none of the participants had deep dyslexia, as indicated by their good reading of nonwords and as indicated by their far better reading of the texts without movement). 

The dyslexias we found in the four participants with dyslexia were: letter position dyslexia, which causes transpositions of letters within words, attentional dyslexia, which causes letter migrations between words, vowel dyslexia, which causes omission, migration, addition or substitution of vowel letters, and orthographic input buffer dyslexia, which causes letter migrations within and between words, omission, migration, and additions of letters. None of these dyslexia types cause word order errors in reading, verb omission, or reading an object marker in an incorrect position in the sentence.

#### 6.3.2. Orally-Trained DHH Children Do Not Necessarily Have a Deficit in the Phonological Representations of Words Causing a Decoding Deficit

A large body of studies ascribed the reading difficulties of orally-trained DHH children to a lack of phonological code of spoken words or to a deficit in phonological representations of words [13,14,18,86].

Our study in fact suggested evidence to the contrary. Firstly, of the 29 DHH children, 22 DHH participants showed good word-level reading, which was not different from age-matched typically hearing controls. Namely, they had no reading difficulty at the word level. Moreover, a phonological difficulty should manifest itself in difficulties in reading new words and nonwords in conversion of letters to phonemes. However, these participants showed nonword reading that was similar to that of hearing controls.

Furthermore, we have evidence of four children who relied even more than hearing controls on the phonological route. The dual route model for single word (and nonword) reading describes two routes for reading aloud: a lexical and a non-lexical route. The lexical route allows reading of familiar words through conversion from a word in an orthographic lexicon to the parallel word in a phonological lexicon [66]. The other route, the non-lexical route, proceeds by conversion from letters to phonemes. Reading through the non-lexical route would result in correct reading of nonwords alongside impaired reading of irregular words (words that cannot be read uniquely and correctly through letter-to-phoneme conversion, such as *stomach, door, night*, and *talk*). The finding that four DHH children showed regularization errors in reading indicates that in fact not only are they able to use the non-lexical route for reading nonwords; they even use it for reading words that they cannot read via the lexical route and their reading of these words indicates correct use of grapheme-to-phoneme conversion rules, which guide reading via the phonological route. This provides strong evidence for their ability to use the phonological representation and the phonological (non-lexical) route in reading. 

These four DHH participants also demonstrate another point of inter-relation between language abilities and reading aloud. We mentioned that the lexical route is built on a connection between an orthographic input lexicon and a phonological output lexicon. If a word is not represented in either of these lexica, reading would have to proceed via the non-lexical route. Indeed, many orally-trained DHH children are reported to have incomplete lexical representation, with fewer words represented in their phonological output lexicon relative to their age [10,68,69], and they often need direct instruction of words in order to enrich their phonological output lexicon. Here, then, their lexical difficulties lead to incorrect (and slower) reading via the non-lexical route. Finally, it may also be the case that their syntactic difficulties, together with their not knowing many of the words, reduce their joy of reading and makes them avoid reading. This, in turn, affects their orthographic input lexicon, causing it to be incomplete, which creates yet another reason for reading via the non-lexical route and for regularization errors. 

### 6.4. The Task Revealed Two Additional Difficult Syntactic Structures

When we created the paragraphs for the reading test, we included sentences with syntactic movement, syntactic structures that we found in our previous studies to be hard for Hebrew-speaking DHH children. The reading errors that the participants eventually made in the paragraphs revealed new domains of difficulty for DHH children: The DHH children made many errors in reading definite determiners and construct state nominals, both very common structures in Hebrew. Friedmann et al. [82] discussed the difficulty in construct state nominals and ascribed it to a deficit in a specific type of head movement (N-to-D movement). The difficulties in reading the definite determiners may have resulted from a syntactic difficulty or from a difficulty with shared knowledge that some studies ascribe to DHH children (for deficits in determiners displayed by DHH children see [87,88]). Future research should examine these difficulties: their source and the way in which they affect comprehension and spoken production.

### 6.5. Educational and Clinical Implications

These results point a strong beam of light on the syntactic abilities of DHH children and their importance in text reading and comprehension, as well as in everyday life. Although DHH children have greatly increased access to sound through early identification and use of sophisticated hearing assistive technology, which allowed them to develop the phoneme system and are using that access to develop decoding skills, many still continue to struggle with complex syntax.

A first measure that can be taken to try and prevent syntactic difficulties altogether is to make sure DHH infants are exposed to language as early as possible, by means of early fitting of hearing devices or rich and consistent exposure to natural sign language. The first year of life is a critical period for the development of syntax in the first language [55,89,90], and missing this critical period may underlie later syntactic difficulties [30,46,47,57,58,91,92].

Additionally, given the preponderance of syntactic difficulties in this population, and especially for those children who did not have sufficient early exposure to language, early assessment of syntactic difficulties is crucial. This requires a battery of syntactic tests for sentences with syntactic movement that are common in the target language and skilled assessors of syntactic abilities.

Once a child is diagnosed with syntactic movement impairment, they can benefit both from teaching that takes these difficulties into account and from treatment that is targeted at the syntactic abilities that are impaired.

Teachers who are aware of the syntactic structures that are especially difficult for DHH children can identify these sentences in the texts, and use other syntactic structures to explain them. They can also work with the child on understanding them by ways of disassembling the complex sentence into its constituents and by rephrasing these sentences to create simple sentences. Such systematic work will pave the way for the pupil to cope with these difficult structures when encountering them in future texts.

DHH children can also benefit from structured syntactic treatment, focusing on syntactic movement (e.g., along the lines of [93,94,95,96]). These intervention programs include explicit instruction of the of structure of sentences derived by syntactic movement: relative clauses, Wh-questions, sentences with topicalization, and sentences with verb movement. In the intervention program, the ability to comprehend and produce these structures is practiced through oral and written assignments.

Our results suggest that syntactic intervention for structures with syntactic movement are expected to affect not only the children’s ability to understand such sentences in isolation but also to contribute to their ability to read and understand texts.

## 7. Conclusions

Studies report that many DHH children show syntactic deficits, other studies report that many DHH children show reading difficulties. Assuming an overlap between these impairments, this could have been taken to be a simple case of co-morbidity between syntactic and reading deficits. However, our results show that this is not the case. Firstly, most DHH children in fact do not have a deficit in reading, as indicated by their age-appropriate reading at the single word level and their good reading of simple sentences. Secondly, this is by no means an accidental co-morbidity: The deficit in syntax is in fact the source of the reading errors that these children show in reading texts that involve syntactically complex sentences.

## Figures and Tables

**Table 1:** Background information on the DHH participants.

Participant	Age	Gender	Age at Diagnosis	Age Hearing Aids Fitted	Age at Implantation	Type of Hearing Loss	Etiology	Hearing Loss (Right, Left) ^a^	Device
DOHM	10;10	male	0;6	1;0		sensorineural	unknown	r-90, l-70	HA
DORF	11;5	female	2;6	3;6		sensorineural	unknown	r-60, l-65	HA
CHBF	9;7	female	0;0	0;2		sensorineural	genetic	r-65, l-70	HA
TABM	9;8	male	0;0	0;6		sensorineural	genetic	r-50, l-50	HA
SHGF	10;6	female	3;0	7;0		sensorineural	unknown	r-85, l-75	HA
SHVM	11;11	male	0;6	1;5		sensorineural	unknown	r-45, l-50	HA
AVCM	10;4	male	0;0	-		sensorineural	genetic	r-85, l-85	HA
IVLM	9;8	male	0;0	3;6		combined	middle ear deformation	r-50, l-50	HA
TSHM	10;10	male	1;4	2;6		sensorineural	genetic	r-80, l-80	HA
KEMF	11;1	female	0;6	3;0		sensorineural	genetic	r-70, l-75	HA
RSHM	10;0	male	0;0	0;9		sensorineural	genetic	r-55, l-55	HA
TAMM	9;8	male	0;3	0;6		sensorineural	premature baby	r-50, l-55	HA
YAOF	10;1	female	3;0	3;0		sensorineural	genetic	r-60, l-65	HA
ROPM	10;9	male	0;3	1;0		sensorineural	genetic	r-50, l-50	HA
NAEF	10;7	female	0;6	0;6		sensorineural	syndrome	r-60, l-65	HA
HIMF	9;11	female	0;7	0;8	1;7	sensorineural	unknown	****	CI
TCHF	11;3	female	0;6	0;10	2;2	sensorineural	syndrome	****	CI
YODM	10;3	male	0;6		1;5	sensorineural	unknown	****	2 CI
NAHM	10;6	male	0;0	0;2	1;0	sensorineural	unknown		2 CI
SHSF	10;6	female	0;0	1;0	2;2	sensorineural	unknown	****	CI
TACF	10;9	female	0;2	0;3	1;0	sensorineural	genetic	****	CI
YIBM	10;3	male	0;9	1;2	1;6	sensorineural	unknown	****	CI
EDYF	9;6	female	1;6	1;9	4;5	sensorineural	genetic	****	CI
LIHF	9;1	female	0;2	0;3	1;0	sensorineural	unknown	****	CI
YAZM	9;11	male	0;0		1;3	sensorineural	genetic	****	2 CI
RARM	11;7	male	0;0	1;0	5;0	sensorineural	unknown	****	CI
LSHM	10;1	male	0;6		1;0	sensorineural	genetic	****	CI
LIAF	10;0	female	0;8		1;3	sensorineural	unknown	****	2 CI
CHBM	12;2	male	0;0	0;6	3;6	sensorineural	genetic	****	CI
LILF	10;10	female	0;0	0;4	5;0	sensorineural	genetic	****	CI
MALF	10;10	female	0;0	0;4	2;6	sensorineural	genetic	****	CI
YCHF	11;5	female	0;0	0;6	1;6	sensorineural	genetic		CI

^a^ All the participants with the CI had a profound congenital hearing loss without the CI; CI = Cochlear Implant, HA = Hearing Aids.

**Table 2:** Performance of each DHH participant in the syntactic tasks (√—not different from hearing controls, ×—significantly below the hearing controls).

	Comprehension				Production			Repetition
Participant	Object Relatives (Test 1)	Object Relatives (Test 2)	Object Questions (Test 2)	Written Object Relatives (Test 3)	Subject Relatives (Test 4)	Object Relatives (Test 4)	Object Relatives (Test 5)	Object Relatives, Object Questions, Topicalizations (Test 6)
DHH with impaired syntactic abilities (LOWSYD)								
LIHF	√	×	√	×	×	×	×	×
YAZM	√	√	×	×	√	√	×	×
AVCM	×	×	×	×	×	√	×	×
HIMF	√	×	√	×	√	√	×	×
RARM	√	√	√	×	√	×	×	×
CHBM	×	×	×	×	×	×	×	×
SHGF	√	√	√	×	√	√	×	×
TSHM	×	×	×	×	√	×	√	√
YAOF	×	×	×	×	√	×	×	√
YODM	√	√	√	×	√	√	×	×
DORF	×	√	×	×	×	√	×	×
NAHM	√	√	√	×	×	√	×	√
IVLM	×			×	√	√	×	×
DOHM	√	√	√	×	×	×	×	√
Marginal or different impairment								
YIBM	√			×	×	√	√	×
SHVM	×			√			√ ^m^	√
LILF	×	×	√	√	√	√	√	√
DHH with intact syntactic abilities (GOODSYD)								
CHBF	√	√	√	√	√	√	×	√
KEMF	√	√	√	√	√	√	√	√
RSHM	√			√	√	√	√	√
NAEF	√			×	√	√	√	√
TAMM	√			√	√	√	√	√
TCHF	√	√	√	√	√	√	√	√
SHSF	√	√	√	√	√	√	√	√
TACF	√	√	√	√	√	√	√	√
EDYF	×			√	√	√	√	√
LIAF	×			√	√	√		√
MALF	√	√	√	√	√	√	√	√
ROPM	√	√	√	√	√	√		√
YCHF	√	√	√	× ^m^	√	√	× ^m^	√
TABM	×	√	√	√	√	√	√	√
LSHM	√			√	√	√	√	×

Empty cells indicate that the participant did not take the test. Shaded cells indicate a performance significantly below the hearing control group. ^m^ Marginal level performance: SHVM produced two grammatical yet non-target responses in the relative production task. YCHF made a single error above the hearing threshold in two tests.

**Table 3:** An example for a pair of matched paragraphs, one with Wh and verb movement and one without. (The text is a translation from Hebrew that retains the original syntactic structures of the Hebrew text. Clauses with Wh- or verb-movement are marked in boldface).

A Paragraph with Wh and V Movement	A Matched Paragraph without WH or V Movement
A trip to Afrika Last year a group of travelers in Africa parked in a small forest for a short rest and lunch. Suddenly **monkeys that the travelers saw** approached them and sat around them. The **travelers that the monkeys surprised** were happy at first, but then **became the monkeys that the travelers photographed rude** and began to take the food from the travelers’ hands. Shouted the travelers at the monkeys and tried to expel them. **The monkeys that the travelers expelled threw stones. The frightened travelers that the monkeys attacked ran away**. **The monkeys that ate everything that the travelers left cheered**.	A trip to Afrika Last year a group of travelers in Africa parked in a small forest for a short rest and lunch. The travelers took pictures of monkeys and suddenly the monkeys approached and sat around the travelers. The bunch of monkeys surprised the group of travelers. The travelers were happy at first but then the monkeys became rude and began to take the food from the traveler’s hands. The travelers shouted at the monkeys and tried to expel them. The monkeys threw stones at the travelers. The monkeys attacked the travelers and the travelers ran away. The travelers left food and the monkeys ate it. The monkeys cheered.

**Table 4:** Mean number (and SD) of reading errors per participant in reading paragraphs with syntactic movement in the two DHH groups and in the hearing control group.

Type of Error	Target Sentence Type	Number of Target Sentences	DHH with Syntactic Impairment (LOWSYD)	DHH with Intact Syntax (GOODSYD)	Hearing Control Group	LOWSYD-Hearing Group Comparison
Structural errors	Object relatives	27	7.8 (6.3)	1.1 (1.1)	1.1 (1.3)	*t* (29) = 4.06, *p* = 0.0002
	Subject relatives	2	0.6 (0.9)	0.2 (0.4)	0.2 (0.6)	*t* (29) = 1.51, *p* = 0.07
	Topicalization	6	0.7 (0.9)	0.5 (0.9)	0.4 (0.5)	*t* (29) = 1.40, *p* = 0.08
	Verb movement	21	1.9 (1.6)	0.9 (1.2)	0.5 (0.9)	*t* (29) = 3.11, *p* = 0.002
	Sentential complements	8	1.4 (1.1)	0.7 (1.2)	0.5 (0.6)	*t* (29) = 3.07, *p =* 0.002
	Simple sentences	5	0.6 (1.0)	0.1 (0.3)	0.2 (0.5)	*t* (29) = 1.65, *p =* 0.05
Function word errors			3.8 (4.3)	1.1 (1.26)	0.7 (0.8)	*t* (29) = 2.93, *p* < 0.003
Lexical errors			2.4 (1.4)	1.1 (1.0)	1.5 (1.3)	*t* (29) = 1.83, *p* = 0.03
Definite determiner			6.8 (3.9)	2.0 (1.9)	1.0 (1.2)	*t* (29) = 5.69 *p <* 0.0001
**Total**			30.8 (19.3)	9.5 (6.3)	6.8 (4.6)	*t* (29) = 4.97, *p <* 0.0001

Shaded cells indicate comparisons that survive Benjamini and Hochberg’s FDR correction.

**Table 5:** Reading errors of each of the DHH children with impaired syntax in the paragraphs with and without movement.

	Reading Errors in Paragraphs with Syntactic Movement									Reading Errors in Paragraphs without Syntactic Movement					
	Structural Errors in:									Structural Errors in:					
participant	Sentences derived by Wh-Movement	Sentences Derived by Verb Movement	Sentential complements	Simple sentences	construct state nominals	Function words	Lexical errors	definite determiner	Total	Sentential complements	Simple sentences	Function words	Lexical errors	definite determiner	Total
LIHF	17	3	2	0	9	8	3	12	54	3	4	2	3	8	20
YAZM	27	3	1	2	12	11	2	17	75	5	11	13	9	16	54
AVCM	5	3	1	0	3	3	3	5	23	0	2	2	1	6	11
HIMF	13	1	1	0	4	1	1	6	27	0	3	0	0	7	10
RARM	6	0	1	0	3	1	2	7	20	3	1	2	0	5	11
CHBM	18	4	4	3	8	13	2	11	63	3	5	3	3	13	27
SHGF	4	0	1	1	4	2	3	4	19	1	0	6	2	8	17
TSHM	8	2	0	0	3	0	3	5	21	0	2	1	1	6	10
YAOF	6	3	3	0	3	0	3	6	24	0	2	0	1	6	9
YODM	8	0	1	0	5	3	3	6	26	0	3	1	2	9	15
DORF	2	1	1	1	3	1	6	3	18	1	2	3	4	4	14
NAHM	8	5	2	2	4	7	1	5	34	2	4	8	0	5	19
IVLM	2	1	0	0	1	0	0	6	10	0	0	0	2	0	2
DOHM	4	1	2	0	3	3	2	2	17	0	2	1	1	3	7
TOTAL	128	27	20	9	65	53	34	95	431	18	41	42	29	96	226

Shaded columns refer to two structures that occur in the paragraphs with movement and not in the paragraphs without movement.

**Figure A1:**
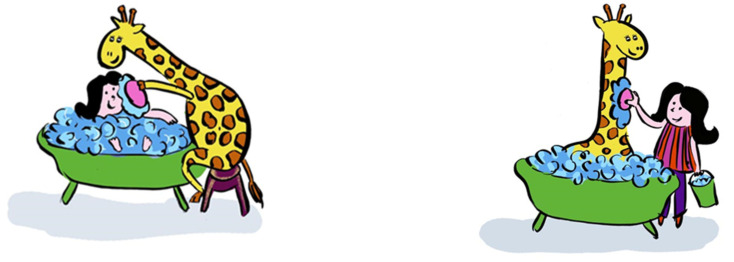
An example of a picture used in the sentence-picture matching task.

**Figure A2:**
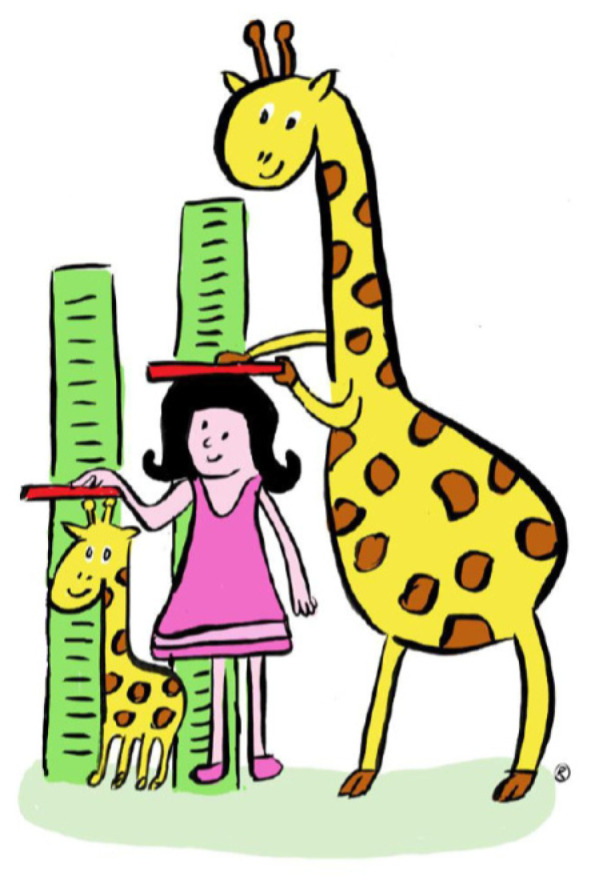
An example of a picture used in the picture selection task.

## Appendix A. Assessment of Syntactic Abilities

Below we report in detail on the six syntactic tests we used to determine, for each DHH child, whether they had a syntactic deficit in movement or not. Table A1 summarizes the performance of the DHH participants and the hearing control group in each syntactic measure and the description of the hearing control group participating in each syntactic test.

### Appendix A.1. Comprehension of Subject and Object Relative Clauses—Sentence-Picture Matching Task

In this task (BAMBI ZXT [97]) each participant heard 40 final-branching subject- and object relative sentences (20 sentences each) while seeing two pictures with two characters each—one picture matched the sentence and the other included the reversed roles (Figure A1). The participant was asked to select the picture matching the sentence. (The test also included 20 coordinated sentences that we do not discuss here [97].) We used the comprehension of the object relative clauses in this task as a measure of the participant’s syntactic abilities.

### Appendix A.2. Comprehension of Relative Clauses and Wh-Questions—A Picture Selection Task

In this task (BAFLA ZST-TLAT [31,98]) each participant heard 80 sentences: subject and object final-branching relative clauses, and subject and object *which* questions, 20 of each type. To create the distractor in which the agent and theme roles are reversed, they were presented with a picture that included three people or animal characters; the first character is performing an action on the second character, and the second character is performing the same action on a third character. The first and third characters were of the same type (both giraffes, both girls, etc.; see Figure A2). The participant was asked to select the character that matched the description in the relative clause or answered the question. We used the comprehension of the object relative clauses and of the object *which* questions in this task as two measures of the participant’s syntactic abilities.

### Appendix A.3. Comprehension of Written Object Relatives: Paraphrasing of Object Relatives with Center Embedding

In this task (BAFLA ZIKRIA [99,100]) the participants were asked to read 20 sentences aloud and then explain them in their own words. Half (10) of the sentences were center-embedded relative clauses, in which the main verbs were heterophonic-homographs of nouns. The other 10 sentences were simple control sentences with the same homographic verbs and the same length. The participants were requested to read each sentence aloud and to paraphrase it as accurately as possible. To reduce demands on memory, each written sentence remained in front of the participants until they finished reading and paraphrasing it (for details about this task see also [46]). We used the paraphrasing of the object relative clauses in this task as a measure of the participant’s syntactic abilities.

### Appendix A.4. Production of Relative Clauses—Elicitation of Relative Clauses in a Picture Description Task

In this task (BAFLA ZIBUV test [101]) we elicited 10 subject relatives and 10 object relatives. The participants saw 10 picture pairs. Each pair of pictures included two characters (people or animals). One picture depicted one character performing an action on the other, and in the second picture, the roles were reversed. The experimenter described the two pictures using simple sentences and then asked about one of the characters and its role in each of the pictures. The target responses were either a subject relative clause or an object relative clause (for details about this task see also [30,102]). We used the production of the subject and object relative clauses in this task as two measures of the participant’s syntactic abilities.

### Appendix A.5. Production of Relative Clauses—Elicitation of Relative Clauses in a Preference Task

In this task (ADIF test [103]), we elicited 10 subject relatives and 10 object relatives. The experimenter described two children in two situations and asked the participant to choose which child s/he would prefer to be. The task was constructed in a way that the choice would have to be formed as a relative clause. To ensure a relative clause response, the experimenter requested the participants to reply to each question starting with “I would rather be…”. The questions that elicited subject relatives described two children performing two different actions on the same theme or performing the same action on two different themes. The questions that elicited object relatives described two children, who are the themes of different actions performed by the same figure, or an action performed by two different figures (see [30,102], for task description). We used the production of the object relative clauses in this task as a measure of the participant’s syntactic abilities.

### Appendix A.6. Sentence Repetition Task

In this task (“PETEL” [104]), the experimenter said a sentence, and the participant was requested to count aloud to three and then repeat the sentence as accurately as possible. When children repeat a sentence in their language, repetition involves the comprehension of the sentence and its reproduction. As a result, a syntactic impairment that affects comprehension and production is manifested in incorrect repetition of the relevant sentences [52]. The test included 70 sentences: 25 sentences with object Wh-movement (10 object relatives, 10 object topicalized sentences, 5 object Wh-questions), 10 sentences with verb movement, and 35 filler sentences without object Wh-movement or verb movement that included the same words as the sentences with movement. All sentences contained four words (object case markers, embedding markers, and prepositions were counted with the word following them). All the sentences derived by Wh-movement were semantically reversible (see [52] for task description). We used the repetition of the object relative clauses, the object questions, and the topicalization structures (repetition without structural errors) in this task as a measure of the participant’s Wh-movement abilities; we used the participants’ ability to repeat the sentences with verb movement as a measure for their verb movement abilities.

**Table A1:** Performance of the syntactically impaired DHH participants and the hearing control group (typically developing hearing Hebrew-speaking children in fourth and fifth grade) in the syntactic tasks.

Task	Hearing Control Group N, Mean Age (SD)	Hearing Control Group Performance	Number of DHH Participants Significantly below the Hearing Controls	Syntactically Impaired DHH Subgroup Performance	Syntactically Impaired DHH vs. Hearing Controls	Syntactically Impaired vs. Syntactically Intact DHH
Comprehension of object relatives—sentence-picture matching task	N = 32 M = 9;11 (0;5)	M = 94.1%, SD = 7.9%	11 (out of 32)	M = 84.3%, SD = 12.2%	*t*(44) = 3.23, *p* = 0.001 *d* = 1.05	*t*(27) = 1.80, *p* = 0.04 *d* = 0.69
Comprehension of object relatives—picture selection task	N = 20 M = 9;9 (0;5)	M = 98.5%, SD = 3.3%	7 (out of 23)	M = 82.7%, SD = 17.6%	*t*(31) = 3.94, *p* = 0.0002 *d* = 1.48	*t*(20) = 2.70, *p* = 0.007 *d* = 1.22
Comprehension of object referential questions—picture selection task	N = 20 M = 9;9 (0;5)	M = 97.5%, SD = 4.1%	6 (out of 23)	M = 85.5%, SD = 15.8%	*t*(31) = 3.24, *p* = 0.001 *d* = 1.19	*t*(20) = 2.03, *p* = 0.027 *d* = 0.92
Comprehension of written object relatives with center embedding—paraphrasing	N = 27 M = 10;0 (0;7)	M = 85.6%, SD = 10.5%	17 (out of 32)	M = 37.1%, SD = 14.4%	*t*(39) = 12.3, *p* < 0.0001 *d* = 4.15	*t*(27) = 7.36, *p* < 0.0001 *d* = 2.8
Production of subject relatives—Elicitation in a picture description Task	N = 18 M = 9;10 (0;7)	M = 98.3%, SD = 3.8%	8 (out of 31)	M = 88.5%, SD = 13.5%	*t*(30) = 2.93, *p* = 0.003 *d* = 1.07	*t*(27) = 2.48, *p* = 0.009 *d* = 0.95
Production of object relatives—Elicitation in a picture description Task	N = 18 M = 9;10 (0;7)	M = 97.7%, SD = 4.2%	5 (out of 31)	M = 78.5%, SD = 24.7%	*t*(30) = 3.24, *p* = 0.001 *d* = 1.19	*t*(27) = 3.23, *p* = 0.001 *d* = 1.24
Production of object relatives—Elicitation in a preference task	N = 36 M = 10;1 (0;7)	M = 93.8%, SD = 8.0%	14 (out of 30)	M = 60%, SD = 28.8%	*t*(48) = 6.52, *p* < 0.0001 *d* = 2.09	*t*(25) = 4.11, *p* = 0.0001 *d* = 1.64
Repetition of sentences derived by Wh-movement	N = 20 M = 9;8 (0;11)	M = 94.6%, SD = 3.7%	12 (out of 32)	M = 72.4%, SD = 19.6%	*t*(32) = 4.97, *p* < 0.0001 *d* = 1.78	*t*(27) = 3.64, *p* = 0.0005 *d* = 1.4
Repetition of sentences derived by verb movement	N = 20 M = 9;8 (0;11)	M = 93.5%, SD = 11.3%	10 (out of 32)	M = 68.9%, SD = 34.5%	*t*(32) = 2.98, *p* < 0.0001 *d* = 1.07	*t*(27) = 1.43, *p* = 0.08 *d* = 0.55

## Appendix B. The TILTAN Reading Task: Properties of the Stimuli and Detailed Individual Results

The TILTAN screening test [63] includes words and nonwords of various types that can reveal impairments in the various components of the word reading process, i.e., the various types of dyslexia. The single word reading part of the test includes 136 words, containing 65 migratable words—words in which middle letter migration creates another existing word, for the identification of letter position dyslexia; 104 words for which omission, substitution, migration, or addition of a vowel letter creates another existing word, for the identification of vowel letter dyslexia; 136 words for which neglect of the left side of the word yields another existing word, for the identification of neglect dyslexia; 108 words for which right neglect errors create an existing word; 84 irregular words and potentiophones for the identification of surface dyslexia; 57 morphologically complex words for deep dyslexia and phonological dyslexia; 26 abstract nouns and 28 function words, for deep dyslexia. All the words were sensitive to errors of letter omission, substitution, or addition and therefore for letter identity/visual dyslexia, as each word had more than six orthographic neighbors.

The 30 nonwords were included for the identification of impairments in the sublexical route, in varieties of phonological dyslexia or vowel dyslexia, and deep dyslexia. All nonwords were such that substitution, omission, or addition of letters created existing words and hence were sensitive to impairments in the orthographic-visual analysis stage; 11 of the nonwords were migratable nonwords and hence sensitive to letter position dyslexia.

The list of 30 word-pairs was created so that each position-preserving letter migration between words created other existing words, for the identification of attentional dyslexia.

**Table A2:** Reading performance in the three parts of the TILTAN reading screening test. √ indicates age-appropriate performance that did not differ from the hearing controls. When performance was significantly below the hearing controls, the cell includes % errors and main error types.

Participant	Words	Nonwords	Word Pairs	Reading at Word Level	Type of Dyslexia
DOHM	√	√	√	intact	
CHBF	√	√	√	intact	
TABM	√	√	√	intact	
SHGF	√	√	√	intact	
IVLM	√	√	√	intact	
TSHM	√	√	√	intact	
KEMF	√	√	√	intact	
RSHM	√	√	√	intact	
NAEF	√	√	√	intact	
TAMM	√	√	√	intact	
YAOF	√	√	√	intact	
ROPM	√	√	√	intact	
EDYF	√	√	√	intact	
TCHF	√	√	√	intact	
YODM	√	√	√	intact	
NAHM	√	√	√	intact	
SHSF	√	√	√	intact	
TACF	√	√	√	intact	
LSHM	√	√	√	intact	
LIAF	√	√	√	intact	
LILF	√	√	√	intact	
MALF	√	√	√	intact	
YCHF	√	√	√	intact	
HIMF	16.9 Regularization errors, sublexical reading	√	5	Impaired	Sublexical reading (surface dyslexia or reduced lexica)
DORF	11.8 Regularization errors, sublexical reading	√	6.7	Impaired	Sublexical reading (surface dyslexia or reduced lexica)
RARM	15.4 Regularization errors, sublexical reading	√	6.7	Impaired	Sublexical reading (surface dyslexia or reduced lexica)
LIHF	29.4 Regularization errors, sublexical reading; letter migrations within and between words; letter and affix omissions and substitutions	50 Migrations within words; letter omissions, substitutions	17 Migrations between words	Impaired	Surface dyslexia and orthographic input buffer impairment
CHBM	17 Migrations within and between words; letter and affix omissions and substitutions	20 Migrations between words	13 Migrations between words	Impaired	Orthographic input buffer impairment
YAZM	36.8 Vowel letter omissions, migrations, substitutions; migrations of letters within and between words	37 Vowel errors and migrations within words	25 Migrations between words	Impaired	Vowel letter dyslexia, attentional dyslexia, letter position dyslexia
AVCM	19.1 Vowel letter omissions, migrations, substitutions; migrations of letters within and between words	23.3 Vowel errors and migrations within words	13.3 Migrations between words	Impaired	Vowel letter dyslexia, attentional dyslexia, letter position dyslexia

## Appendix C. The Types of Sentence and Question Types in the Paragraphs

**Table A3:** The types of structures included in the 6 paragraphs with movement, the number of sentences of each type, and examples.

Type of Structure	Number of Sentences	An Example from the Paragraphs
**Sentences Derived by Wh-movement**		
Object relative—center embedding	22	The monkeys that the travelers expelled threw stones.
Object relative—final embedding	3	Yoad said that a short figure that his mother saw in the dark disappeared quickly without a trace.
Object topicalization	6	Acc these boys, the policemen couldn’t see.
PP object relative	2	And in one large cage that-in-it a pool…
Subject relative—center embedding	2	But sometimes there are brave birds that insist on penetrating the robin’s territory.
**Sentences with Verb Movement**		
A simple sentence with verb movement	15	One day went the second graders on a trip to the zoo.
Verb movement in a relative clause (in the main clause or the embedded clause)	6	but then became the monkeys that the travelers photographed rude.
**Sentences without Movement**		
A simple sentence	4	Last year a group of travelers in Africa parked in a small forest for a short rest and lunch.
Sentence embedding to a verb	10	Daniel said that his parents came home late the previous night.

**Table A4:** Types of comprehension questions.

Type of Question	Number of Questions	An Example for a Question
Subject question—Who	16	Who threw stones?
Object question—What	5	What did the travelers photograph?
Adjunct question—When/Why/How	4	Why did the neighbors go to the street in the evening?
Object question—Whom, with arbitrary pro subject	3	Whom did they push? (et mi daxfu? = acc who arb-pro push)

## Appendix D. Examples for Errors in Reading Sentences with Movement

### Appendix D.1. Reading Errors in Object Relatives

(1) Omission of an obligatory relativizer.

Target sentence:
  Be-kluv gadol exad ra’u ha-yeladim zebra **she**-jirafa daxafa
  In-cage big one, saw the-children zebra **that**-giraffe pushed
  In one large cage, the children saw a zebra **that** a giraffe pushed.
Incorrect reading:
  *Be-kluv gadol exad ra’u ha-yeladim zebra jirafa daxafa
  In-cage big one, saw the-children zebra giraffe pushed
  In one large cage, the children saw a zebra a giraffe pushed.

(2) Omission of the main verb.

Target sentence:
  cevet kibuy esh she-toshav ha-shxuna hizmin **hegia** im sulam be-orex 30 metrim
  squad extinguishing fire that-resident the-neighborhood invited **came** with ladder in-length 30 m
  A firefighter squad that one of the neighborhood residents invited **came** with a 30-m ladder.
Incorrect reading:
  *Cevet kibuy esh she-toshav ha-shxuna hizmin im sulam be-orex 30 metrim.
  squad extinguishing fire that-resident the-neighborhood invited with ladder in-length 30 m
  A firefighter squad that one of the neighborhood residents invited with a 30-m ladder.

(3) Incorrect reading of the verb in the relative clause (as a noun).

Target sentence:
  Kvucat ha-metaylim she-xavurat ha-kofim **hifti’a** samxa ba-hatxala
  group the-hikers that-troop the-monkeys **surprised** rejoiced in-the-beginning
  The hikers’ group that the monkey troop **surprised** was happy at first.
Incorrect reading:
  *Kvucat ha-metaylim she-xavurat ha-kofim **hafta’a** samxa ba-hatxala
  group the-hikers that-troop the-monkeys **a-surprise-noun** rejoiced in-the-beginning
  The hikers’ group that the monkey troop **a surprise** was happy at first.

(4) Addition of a coordination marker before the verb of the main clause.

Target sentence:
  Ha-dov ha-gadol she-ha-pil hirtiv ka’as meod
  The-bear the-big that-the-elephant sprayed became-angry very
  The big bear that the elephant sprayed was very angry.
Incorrect reading:
  *Ha-dov ha-gadol she-ha-pil hirtiv **ve-**ka’as meod
  The-bear the-big that-the-elephant sprayed **and** became-angry very
  The big bear that the elephant sprayed **and** was very angry.

### Appendix D.2. Reading Errors in Sentences with Topicalization

(1) Omission of the object case marker before the topicalized object, the object becomes subject.

Target sentence:
  … ax **et ha-kofim** ve-ha-nemerim ahavu ha-yeladim yoter mi-kol
  but **ACC the-monkeys** and-the-tigers loved the-children more than-anything
  But the children loved the monkeys and tigers more than anything else.
Incorrect reading:
  *… ax **ha-kofim** ve-ha-nemerim ahavu ha-yeladim yoter mi-kol
  but **the-monkeys** and-the-tigers loved the-children more than-anything

(2) Addition of an object case marker before the post-verbal subject.

Target sentence:
  … ax et ha-kofim ve-ha-nemerim ahavu ha-yeladim yoter mi-kol
  but ACC the-monkeys and-the-tigers loved the-children more than-anything
  But the children loved the monkeys and the tigers more than anything else.
Incorrect reading:
  *… ax et ha-kofim ve-ha-nemerim ahavu **et** ha-yeladim yoter mi-kol
  but ACC the-monkeys and-the-tigers loved **ACC** the-children more than-anything.

### Appendix D.3. Reading Errors in Sentences with Verb Movement

(1) Omission of the post-verbal subject in a sentence with verb movement.

Target sentence:
  u-ve-kluv gadol axer she-be-toxo breyxa, ra’u **ha-yeladim** shney dubim ve-pil
  and-in-cage big another that-in-him pool saw **the-children** two bears and-elephant
  And in another large cage with a pool in it, **the children** saw two bears and an elephant.
Incorrect reading:
  *u-be-kluv gadol axer she-be-toxo breyxa, ra’u shney dubim ve-pil
  and-in-cage big another that-in-him pool saw two bears and-elephant
  And in another large cage with a pool in it, saw two bears and an elephant.

(2) Addition of an object case marker to the post-verbal subject in a sentence with verb movement where the subject becomes an object.

Target sentence:
  Kibel ha-nasix et ha-hazmana, higia la-kirkas ve-cafa ba-hofa’a
  Got the-prince ACC the-invitation came to-the-circus and-watched in-the-show
  The prince got the invitation, came to the circus and watched the show.
Incorrect reading:
  *Kibel **et** ha-nasix et ha-hazmana higia la-kirkas ve-cafa ba-hofa’a
  received **ACC** the-princess ACC the-invitation came the-circus and-watched in-the-show
